# Cyclic ADP-Ribose and Heat Regulate Oxytocin Release via CD38 and TRPM2 in the Hypothalamus during Social or Psychological Stress in Mice

**DOI:** 10.3389/fnins.2016.00304

**Published:** 2016-07-22

**Authors:** Jing Zhong, Sarwat Amina, Mingkun Liang, Shirin Akther, Teruko Yuhi, Tomoko Nishimura, Chiharu Tsuji, Takahiro Tsuji, Hong-Xiang Liu, Minako Hashii, Kazumi Furuhara, Shigeru Yokoyama, Yasuhiko Yamamoto, Hiroshi Okamoto, Yong Juan Zhao, Hon Cheung Lee, Makoto Tominaga, Olga Lopatina, Haruhiro Higashida

**Affiliations:** ^1^Department of Basic Research on Social Recognition and Memory, Research Centre for Child Mental Development, Kanazawa UniversityKanazawa, Japan; ^2^Department of Biochemistry and Molecular Vascular Biology, Kanazawa University Graduate School of Medical SciencesKanazawa, Japan; ^3^Department of Biochemistry, Tohoku University Graduate School of MedicineSendai, Japan; ^4^School of Chemical Biology and Biotechnology, Peking University Graduate SchoolShenzhen, China; ^5^Division of Cell Signaling, Okazaki Institute for Integrative Bioscience (National Institute for Physiological Sciences), National Institutes of Natural SciencesOkazaki, Japan; ^6^Research Institute of Molecular Medicine and Pathobiochemistry, Krasnoyarsk State Medical UniversityKrasnoyarsk, Russia

**Keywords:** oxytocin, secretion, hyperthermia, NAD, cyclic ADP-ribose, stress, autism

## Abstract

Hypothalamic oxytocin (OT) is released into the brain by cyclic ADP-ribose (cADPR) with or without depolarizing stimulation. Previously, we showed that the intracellular free calcium concentration ([Ca^2+^]_i_) that seems to trigger OT release can be elevated by β-NAD^+^, cADPR, and ADP in mouse oxytocinergic neurons. As these β-NAD^+^ metabolites activate warm-sensitive TRPM2 cation channels, when the incubation temperature is increased, the [Ca^2+^]_i_ in hypothalamic neurons is elevated. However, it has not been determined whether OT release is facilitated by heat *in vitro* or hyperthermia *in vivo* in combination with cADPR. Furthermore, it has not been examined whether CD38 and TRPM2 exert their functions on OT release during stress or stress-induced hyperthermia in relation to the anxiolytic roles and social behaviors of OT under stress conditions. Here, we report that OT release from the isolated hypothalami of male mice in culture was enhanced by extracellular application of cADPR or increasing the incubation temperature from 35°C to 38.5°C, and simultaneous stimulation showed a greater effect. This release was inhibited by a cADPR-dependent ryanodine receptor inhibitor and a nonspecific TRPM2 inhibitor. The facilitated release by heat and cADPR was suppressed in the hypothalamus isolated from CD38 knockout mice and CD38- or TRPM2-knockdown mice. In the course of these experiments, we noted that OT release differed markedly between individual mice under stress with group housing. That is, when male mice received cage-switch stress and eliminated due to their social subclass, significantly higher levels of OT release were found in subordinates compared with ordinates. In mice exposed to anxiety stress in an open field, the cerebrospinal fluid (CSF) OT level increased transiently at 5 min after exposure, and the rectal temperature also increased from 36.6°C to 37.8°C. OT levels in the CSF of mice with lipopolysaccharide-induced fever (+0.8°C) were higher than those of control mice. The TRPM2 mRNA levels and immunoreactivities increased in the subordinate group with cage-switch stress. These results showed that cADPR/CD38 and heat/TRPM2 are co-regulators of OT secretion and suggested that CD38 and TRPM2 are potential therapeutic targets for OT release in psychiatric diseases caused by social stress.

## Introduction

Oxytocin (OT) is preferentially released in response to emotional, physical, and pharmacological stresses (Ebner et al., 2005; Brunton and Russell, 2008; Neumann and Landgraf, 2012; Hashimoto et al., 2014; Kirsch, 2015; Leng et al., 2015; Neumann and Slattery, 2016; Shamay-Tsoory and Abu-Akel, 2016). OT can exert profound anxiolytic and antistress effects in the brain and modulates plasma adrenocorticotropic hormone and corticosterone levels (Quirin et al., 2011; Feldman et al., 2016; Neumann and Slattery, 2016). Therefore, in the central nervous system, OT is considered to act as an anxiolytic factor against stress (Onaka et al., 2012). However, the molecular mechanisms underlying how brain OT is released during stress and the time sequence of OT release after stressful stimulation are unclear.

There have been a number of previous reports of the temperature sensitivity of OT release. It has been shown that endotoxin and interleukin-1 beta induce fever and increase plasma OT levels in rabbits (Hansen and Christensen, 1992), and that OT is released when the body temperature is increased by prostaglandin in rats (Landgraf et al., 1990). OT is released in a nitric oxide-dependent manner during endotoxemic shock (Stabile et al., 2010) or lipopolysaccharide (LPS) treatment (Borges and da Rocha, 2006) in rats. Thus, the involvement of OT in inflammatory reactions and fever is well documented (Landgraf et al., 1990; Hansen and Christensen, 1992; Butterweck et al., 2003; Borges and da Rocha, 2006), but the molecular mechanisms underlying how brain OT is released during hyperthermia are not clear.

CD38, a type II transmembrane glycoprotein with ADP-ribosyl cyclase activity (Jin et al., 2007; Zhao et al., 2012; Kim, 2014; Okamoto et al., 2014), is expressed at high levels in both the mouse and human hypothalamus (Jin et al., 2007; Munesue et al., 2010). The hypothalamic ADP-ribosyl cyclase component of CD38 is activated by OT receptor stimulation, which facilitates the catalytic activity of the cyclic ADP-ribose (cADPR) from β-NAD^+^ (Lopatina et al., 2010). cADPR induces Ca^2+^ release through ryanodine Ca^2+^ release channels from cADPR-sensitive intracellular Ca^2+^ pools, thereby increasing the intracellular free Ca^2+^ concentration ([Ca^2+^]_i_). Such CD38-dependent and cADPR-sensitive [Ca^2+^]_i_ increases likely facilitate OT secretion into the brain mainly from dendrites or axons even in the absence of depolarization in oxytocinergic neurons (Jin et al., 2007; Higashida, 2016). This release seems to be sensitive to β-NAD^+^ and ADPR, but less sensitive to nicotinic acid adenine diphosphate (NAADP) (Jin et al., 2007), suggesting the involvement of β-NAD^+^ metabolites but not NADH metabolites in the CD38-dependent manner.

In contrast, it is well known that β-NAD^+^ metabolites target several ion channels in warmth-sensitive neurons in the preoptic area or anterior hypothalamus, and play an important role in thermoregulation (Nakayama, 1985; Tominaga and Caterina, 2004; Morrison and Nakamura, 2011). One such channel is the transient receptor potential melastatin 2 (TRPM2, previously known as TRPC7 or LTRPC2) (Tominaga and Caterina, 2004; Uchida and Tominaga, 2011; Baez et al., 2014; Faouzi and Penner, 2014; Kashio and Tominaga, 2015). TRPM2 is a member of the warmth-sensing family, and the activation of TRPM2 non-specific cation channels results in Ca^2+^ influx in response to warm temperatures from 34°C to 40°C, which are within the body temperature range of mammals (Perraud et al., 2001; Uchida and Tominaga, 2011). TRPM2 channels can be activated by β-NAD^+^, ADP-ribose (ADPR), and cADPR (Perraud et al., 2001; Uchida and Tominaga, 2011; Baez et al., 2014). Therefore, we hypothesized that OT release is potentially facilitated by activation of TRPM2 channels. This is feasible, because a type of heat sensitivity similar to that found in TRPM2 or [Ca^2+^]_i_ sensitivity in cADPR was reported previously during insulin secretion from pancreatic β cells (Takasawa et al., 1993; Togashi et al., 2006; Uchida et al., 2011).

To assess this possibility, we measured [Ca^2+^]_i_ in acutely cultured hypothalamic cells, and showed that the increases in [Ca^2+^]_i_ in hypothalamic cells are cADPR- and ADPR-dependent and warmth-sensitive in a manner that is susceptible to 2-aminoethoxydiphenyl borate (2APB), a nonspecific TRPM2 Ca^2+^ influx channel inhibitor (Liu et al., 2012). These findings suggested that the TRPM2 cation channel and CD38 are simultaneously involved in heat-potentiated and β-NAD^+^ metabolite-sensitive [Ca^2+^]_i_ increases in oxytocinergic neurons (Amina et al., 2010; Liu et al., 2012). Next, we measured OT release, as no previous studies examined whether OT secretion is dependent on TRPM2 channels.

In the present study, we examined whether CD38 and TRPM2 are involved in triggering OT release from the acutely cultured mouse hypothalamus by heat stimulation and external cADPR application with or without a TRPM2 channel or ryanodine receptor inhibitor in wild-type and CD38 knockout mice. We also examined the time course of changes in OT concentrations in the incubation medium at 3-min intervals in one cultured hypothalamus from each group-housed wild-type male mouse of the ICR strain. The time courses of OT release with the two stimuli were also examined in the hypothalami from CD38- and TRPM2-knockdown mice transfected with specific siRNAs.

Initially, we expected that there would be significant increases with these cofactors equally in all mice. Unexpectedly, we detected responsive and non-responsive hypothalami to incubation with 100 μM cADPR and a shift in incubation temperature from 35°C to 38.5°C. In the majority of mice examined (69.6%, *N* = 46), the OT level did not increase markedly. During these experiments, we noted that OT secretion varied markedly among individuals in group-housed mice with or without injuries, suggesting that maintaining male mice in the group house causes strong stress and forms social hierarchy from ordinate to subordinate mice (Long et al., 1990; Rasmussen et al., 2011). To obtain more direct evidence regarding differential OT release in the same two classes of stress-treated mice, we performed brain microperfusion experiments and measured OT concentrations in microperfusates (extracellular fluids) from the hypothalamus.

To clarify the relationship between OT release and heat under stress conditions *in vivo*, we used two different stress conditions that are known to elevate body temperature: the open field test (anxiety stress due to new environment; LeMay et al., 1990; Lopatina et al., 2014); and the lipopolysaccharide (LPS)-induced fever model (Yirmiya et al., 2001). We measured rectal temperature and OT concentrations in the cerebrospinal fluid (CSF) in both models. Finally, to explain facilitated OT release in subordinate mice with social stress at the molecular level, we examined CD38 and TRPM2 expression levels in the hypothalamus by measuring mRNA levels and CD38 and TRPM2 immunoreactivities.

Although these experiments began almost 8 years ago, the physiological relevance and importance of OT release facilitated by hyperthermia and stress has not been clarified. However, Norton (2014) reported that a single dose of suramin, a century-old drug for African sleeping sickness, eliminated autism symptoms in adult mice with an experimental form of the disorder (Naviaux et al., 2015), and in 2007 it was reported that 83% of children with autism spectrum disorders (ASDs) showed temporary improvement during high fever (Curran et al., 2007), prompting us to complete our experiments. Here, we discuss our findings regarding hyperthermia-induced OT release in the context of clinical case reports of behavioral improvement in children with ASDs associated with fever (Curran et al., 2007; Good, 2011, 2013; Megremi, 2013; Naviaux et al., 2015).

## Materials and methods

cADPR used was purified as described by Lee et al. (1997). ADPR, 8-bromo-cADPR, β-NAD^+^, ryanodine, LPS, 2-aminoethoxydiphenyl borate (2-APB), and OT were purchased from Sigma Chemical Co. (St. Louis, MO, USA). Taq polymerase was obtained from Takara Biomedicals (Otsu, Japan).

### Mice

Slc:ICR (CD-10) outbred male mice (10–12 weeks old, 30–35 g body weight) were obtained from Japan SLC Inc. (Hamamatsu, Japan) via a local distributor (Sankyo Laboratory Service Corporation, Toyama, Japan). In over half of the experiments, the offspring of ICR mice were bred in our laboratory colony, weaned at 25–30 days of age, and housed in same-sex groups of 3–5 animals. In general, 4–5 males were kept in one cage in the animal center under standard conditions (24°C; 12/12-h light/dark cycle, with lights on at 8:45 a.m.) with food and water *ad libitum*. CD38 KO mice were maintained as described previously (Kato et al., 1999; Jin et al., 2007).

To obtain mice with local CD38 and TRPM2 knockdown, the mice were anesthetized with pentobarbitone sodium (65 mg/kg intraperitoneally, diluted 1:10 in sterile saline) and covered with a cotton cloth to maintain normal body temperature in the surgery room at 25°C. The mice were placed securely in a stereotaxic apparatus (Narishige Instrument Inc., Tokyo, Japan) where the skull level was between bregma and lambda. The stereotaxic coordinates were determined from the standard atlas of the mouse brain reported by Franklin and Paxinos (2008), and they were set for the third ventricle: LR 0 mm, AP 0.7 mm, DV 4.2 mm from bregma. Next, 1.0 × 10^6^ infectious units of virus (IFU) containing CD38 shRNA (m) lentiviral particles (sc-37246-v, Santa Cruz Biotechnology Inc., Santa Cruz, CA, USA) or TRPM2 shRNA (m) lentiviral particles (SC-42675-v, Santa Cruz Biotechnology Inc.) were dissolved in 200 μl Dulbecco's modified Eagle's medium with 25 mM HEPES, pH 7.3. The shRNA solution (5 μl) was microinjected into the third ventricle at a perfusion rate of 0.2 μl/min for 25 min with an automated injector and the needle was left for an additional 10 min before it was withdrawn. The mice were used 2 weeks after recovery and effective infection with lentiviruses.

All of the animal experiments were conducted in accordance with the Fundamental Guidelines for Proper Conduct of Animal Experiment and Related Activities in Academic Research Institutions under the jurisdiction of the Ministry of Education, Culture, Sports, Science and Technology of Japan, and they were approved by the Committee on Animal Experimentation of Kanazawa University.

### Social dominance tube test and stress paradigm

The tube test apparatus comprised a 30-cm, smooth, transparent acrylic tube with an internal diameter of 3.5cm. Two mice were positioned at opposite ends of the tube and released simultaneously. Losers were the animals that retreated from the tube, where a full retreat was determined by the absence of any paws within the tube. Next, the two mice were paired and housed together. The social range determined on the first day was maintained or strengthened by psychological stress with a paradigm known as cage-switch stress (Long et al., 1990; Rasmussen et al., 2011). This stress was given by placing mice in an empty clean cage every day at around 9:30 a.m. The rank was unchanged after 4 days. Exposure to the olfactory and visual stimuli associated with this new environment caused a temperature elevation of ~1°C (from 36.1 ± 0.2°C on the first day to 37.1 ± 0.4°C after 4 days, *N* = 5, *P* < 0.01, two-tailed Student's *t*-test) in subordinate mice but there was no increase in ordinate mice (from 36.4 ± 0.2°C to 36.3 ± 0.4°C, *N* = 5).

### OT release from the hypothalamus

CD38^+∕+^, CD38^−∕−^, or CD38, and TRPM2 knockdown mice were anesthetized with pentobarbitone sodium at a dose of 50 mg/kg. One whole hypothalamus was obtained and placed in a 24 multi-well dish plate with 0.4 ml normal Locke's solution containing (in mM): NaCl, 140; KCl, 5; MgCl_2_, 1.2; CaCl_2_, 2.2; glucose, 10; HEPES, 10; bovine serum albumin (BSA), 0.01% adjusted to pH 7.25 with Tris-HCl in a water bath at 35°C. The incubation medium was replaced 10 times every 3 min. After the 11th replacement, the aliquots were retained following a 3-min incubation with the hypothalamus. cADPR was applied to the medium from the 12th replacement. From the 14th replacement, the temperature was shifted to 38.5°C. In addition, 8-bromo-cADPR or 2-APB was applied from the 10th replacement and aliquots were retained from the 8th replacement. Alternatively, the temperature shift was applied from the 11th replacement and cADPR was applied to the medium from the 14th replacement. After 12 extensive washes, OT levels in the incubation medium were almost constant from the 12th to 18th wash; at the 18th replacement, the level was 1.04 ± 0.11-fold that seen at the 12th replacement (*N* = 5).

### Enzyme immunoassay for OT

The OT immunoreactivity levels were quantified using an OT EIA kit (Assay Design, Ann Arbor, MI and Enzo Life Sciences, NY, USA) without pretreatment, as described previously (Jin et al., 2007). The CSF samples (5 μl) were thawed and diluted 1:20 in assay buffer. The plasma samples (100 μl) were thawed on ice and assayed without dilution by the Assay Design's kit and with 1:20 dilution by the Enzo's kit. The OT assay had a sensitivity of 5 pg/ml and the inter- and intra-assay coefficients of variation were < 15%.

### Microperfusion

To implant the microperfusion probe, the mice were anesthetized via a subcutaneous injection of ketamine. The head was fixed in a stereotactic frame (Narishige, Tokyo, Japan) and the mouse was prepared for surgery by shaving its head and disinfecting the skin with 70% ethanol. A spherical dental drill was used to drill a 1-mm hole in the skull while leaving the dura intact. The dura was then punctured with fine forceps to create a defined opening in the meninges. Using the stereotactic frame, a healing dummy was inserted slowly into the three brain positions. The probe was fixed to the skull using two anchor screws and biocompatible dental cement. All of the surgical procedures were completed within 30 min. A healing dummy was used to provide mechanical stability during implantation and throughout the healing period of 2 weeks. The microperfusion probe (a 4-mm length of coaxial tube, 2.5 mm in diameter) comprised a 20-G fluorinated ethylene propylene guide cannula and it was replaced before sampling with in- and out-flow tubing on the day of the experiments. This tubing was connected to two glass syringes (Hamilton, USA) placed in syringe pumps (Eicom, Osaka, Japan). Microperfusate was pumped into the probe at a flow rate of 2 μl/min and the samples were withdrawn at the same flow rate. Sampling was conducted for 2 h. Both microprobes were perfused without sampling for 60 min before the first 30-min microperfusates from the PVN were collected. The microperfusate was mixed under sterile conditions and it comprised 154 mM NaCl, 2.2 mM CaCl_2_, 5.6 mM KCl, 2.3 mM NaHCO_3_, and 0.15% BSA (pH 7.4). Immediately after the application of cADPR, four additional microperfusates were taken at 30-min intervals. After the termination of the experiments, the brains were removed and snap-frozen to obtain 40-μm cryo-cut stained brain slices, which were used later for histological verification of the perfusion site.

### Open field test

The open field test measures locomotion and anxious behaviors, as described previously (Lopatina et al., 2014). The open field apparatus comprised a square box (600 × 600 × 200 mm) lined with polypropylene sheets inside the wooden box. The center arena (300 × 300 mm) was outlined. Each animal was placed in the box for 10 min. The overall activity was measured in the box, and the amount of time and the distance traveled in the center arena were noted. The distance traveled in the field was recorded using a digital video system and ANY-maze software (Liu et al., 2013). This paradigm is based on the idea that mice will naturally prefer to be located near a protective wall rather than being exposed to danger in the open space. After each trial, the test chambers were cleaned with a damp towel and 1% sodium hypochlorite followed by 70% ethanol (Zhong et al., 2014).

### CSF and blood sampling

CSF was collected according to the protocol described for sampling CSF from mice without detectable plasma contamination (Fleming et al., 1983: Liu and Duff, 2008). Briefly, mice were intraperitoneally anesthetized with sodium pentobarbital (50 mg/kg). The skin was shaved on the neck and a sagittal incision was made in the skin inferior to the occiput. The subcutaneous tissue and muscles were separated. The dura mater of the cisterna magna appeared as a glistening and clear reverse triangle through which the medulla oblongata, a major blood vessel (arteria dorsalis spinalis), and the CSF space was visible. A capillary tube was inserted into the cisterna magna through the dura mater, and samples were collected with a 1-ml syringe with a 26-G needle. The CSF was frozen immediately on dry ice and then transferred to a −80°C freezer. After CSF sampling, the heart was exposed, and a blood sample was drawn with a 1-ml syringe. The plasma was centrifuged immediately at 1500 × *g* for 10 min and then stored at −80°C.

### Measurement of body temperature

The rectal temperature was measure by inserting a thermistor probe up to a length of 2 cm in the mouse rectum. Digital recordings of the temperature were obtained with an accuracy of 0.1°C using a digital thermometer (model NS-TC10, Neuroscience Inc., Tokyo, Japan). The probe was dipped into silicon oil before insertion and held in the rectum until a stable rectal temperature was measured for 5 s. The mouse was handled near the base of the tail.

### Real-time PCR

Total RNA was extracted from ICR mouse brain tissues using an RNeasy Lipid Tissue Mini Kit (74804, Qiagen Science, MA, USA), according to the manufacturer's instructions, and reverse transcribed into cDNA using a SuperScript™ First-Strand Synthesis System for RT-PCR (11904-018, Invitrogen, Carlsbad, CA, USA). The cDNA was used as the template for real-time PCR analysis, where the reactions were performed with a ViiA™7 Real-Time PCR System (Applied Biosystems, Foster City, CA, USA). Each sample was assayed in triplicate in a 20 μl amplification reaction mixture containing 10 μl FAST qPCR MasterMix Plus (315-81021, Eurogentec, Seraing, Belgium), 1 μl TaqMan Gene Expression Assay (CD38, Mm01220906_m1; TRPM2, Mm00663098_m1, Applied Biosystems), 2 μl cDNA template, and 7 μl nuclease-free water on a MicroAmp Fast 96-well reaction plate. The values obtained for the genes were normalized against GAPDH mRNA (Mm99999915_g1, Applied Biosystems) expression. Quantitative real-time PCR was performed using a ViiA™7 Real-Time PCR System based on the relative standard curve method, where the relative changes in gene expression by the target were normalized against GAPDH. The quantitative PCR efficiencies were determined by a series of 1:5 dilutions for each experiment.

### CD38, TRPM2, and OT immunostaining

Immunohistochemistry for CD38, TRPM2, and OT were performed as described previously (Jin et al., 2007). Briefly, anesthetized mice were perfused intracardially with cold PBS followed by cold 4% paraformaldehyde (PFA) in PBS. The brains were removed and post-fixed overnight in a 4% PFA solution at 4°C. The brain regions were cut into 2–4 large blocks. The blocks were then sliced on a microtome into 20-μm-thick sections. The sections were pre-incubated in blocking solution (3% BSA and 0.3% Triton X-100 in PBS) for 1 h, and then incubated with a rabbit polyclonal antibody to mouse CD38 (sc-7049, Santa Cruz Biotechnology Inc.), a rabbit polyclonal antibody to rat TRPM2 (C-terminus) (LS-C141843, LifeSpan BioScience, Seattle, WA, USA), and a mouse monoclonal antibody to mouse OT (PS38, ATC CRL 1950) in the blocking solution for 12 h at 4°C. After three washes with washing buffer, the sections were incubated with goat anti-rabbit IgG antibody coupled with Alexa Fluor 488 (Invitrogen) in the blocking solution for 1 h at room temperature. Images were obtained using an Olympus IX71 inverted microscope equipped with a cooled CCD camera (Cool SNAP HQ2; Roper Scientific, Tucson, AZ, USA). The number of immuno-positive nuclei in each brain section were recorded and analyzed using Metamorph software (Molecular Devices, Downingtown, PA, USA).

### Statistical analyses

All of the results were expressed as the mean ± SEM. Two-tailed Student's *t*-tests and one- or two-way ANOVA followed by Bonferroni *post hoc* tests were used to analyze data with unequal variances between groups. In all of the analyses, *P* < 0.05 indicated significant differences.

## Results

It has been shown that cADPR extracellularly applied exerts as an intracellular second messenger and facilitates OT release without depolarizing stimulation (Jin et al., 2007). However, it has not benn demonstrated yet that heat or a combination of heat and cADPR display facilitated OT release from the isolated hypothalamus. Therefore, to assess whether OT can be released from the hypothalamus by two factors, *i.e.*, cADPR and heat *in vitro*, we used the previous paradigm of culture system of the hypothalamus (Jin et al., 2007; Liu et al., 2013). We measured the OT concentrations in the incubation medium of cultured hypothalamic tissue, which was acutely dissected from adult male mice of wild-type (CD38^+∕+^) or CD38 knockout (KO, CD38^−∕−^) mice that belonged to the ICR (CD-10) outbred strain.

### OT release is stimulated by cyclic ADP-ribose and heat *in vitro*

**Figure 1:**
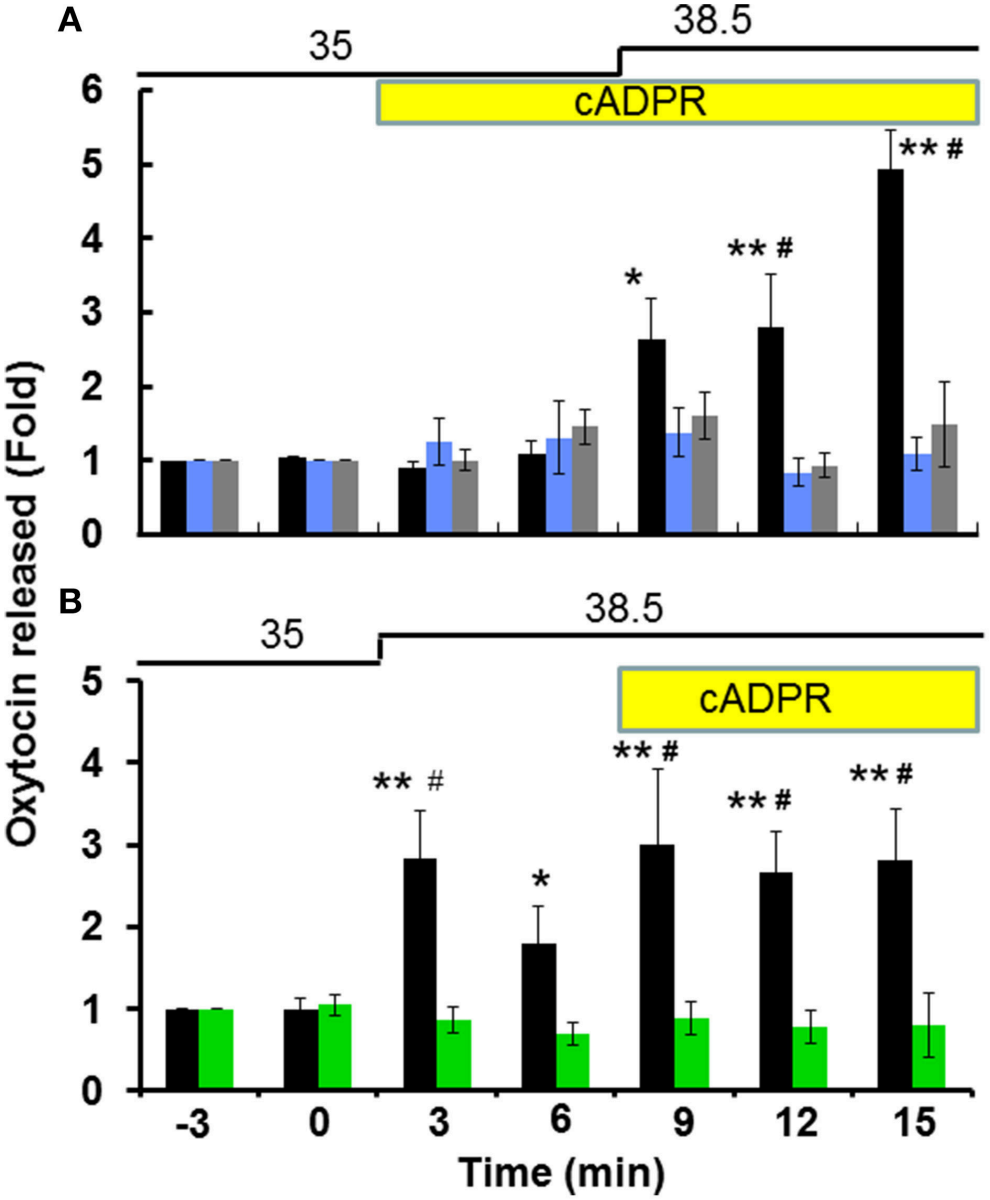
**Oxytocin (OT) release from the isolated hypothalamus**. Whole hypothalamus of group-housed (social stress) mice belonging to both the CD38^+∕+^ or CD38^−∕−^ strains were organo-cultured as described in the Section Materials and Methods. The OT concentrations released from one whole hypothalamus into the medium in one well were measured every 3 min. Stimulation with 100 μM cADPR (yellow bar) and a temperature shift of 3.5°C from 35 to 38.5°C (thick line) are indicated. Data are shown as fold changes in the OT levels relative to 6 min before the start of stimulation as 1.0. **(A)** Application of cADPR followed by heat stimulation in CD38^+∕+^ mice. Filled bars represent data without any inhibitors. One-way ANOVA, *F*_(6, 21)_ = 26.70, *P* < 0.0001: Bonferroni's *post hoc* tests, ^*,**^P < 0.05 and 0.01 from the basal level, respectively. In some experiments, 100 μM 8-Br-cADPR (blue) or 10 μM 2APB (gray) were present in the incubation medium from 20 min before and during the stimulation. Two-way ANOVA, *F*_(12, 78)_ = 6.40, *P* < 0.0001. Bonferroni's *post hoc* tests detected significant differences between the cADPR and heat vs. 2-APB (^#^*P* < 0.01) and 8-Br-cADPR (^#^*P* < 0.01) treated groups at 12 and 15 min. **(B)** Application of heat followed by cADPR. Filled and green bars represent data from CD38^+∕+^ and CD38^−∕−^ mice, respectively. *N* = 16, one-way ANOVA, *F*_(4, 200)_ = 30, *P* < 0.01 in CD38^+∕+^ mice: Bonferroni *post hoc* tests, ^*,**^P < 0.05 and 0.01 from the basal level, respectively. Two-way ANOVA, *F*_(6, 56)_ = 3.18, *P* < 0.01. The difference between the two genotypes was significant *F*_(1, 56)_ = 40.07, *P* < 0.0001. The data obtained with the CD38^−∕−^ mice significantly differed (^#^*P* < 0.01). The initial value (1.0) at the 12th replacement refers to the concentrations in the incubation medium of 17.8 ± 2.6 pg/ml (*N* = 30).

Figure 1A illustrates the time course of the OT concentrations in the incubation medium at 3 min intervals in one hypothalamus from the group-housed wild-type mice. Incubation with 100 μM cADPR alone induced no or minor increases in the OT concentration in the culture medium, but the OT concentration increased significantly with additional heat stimulation. When the incubation temperature was increased from 35 to 38.5°C, the OT concentration increased by 2.6 ± 0.27-fold and 4.1 ± 0.46-fold (*N* = 14) compared with the pre-stimulation level after 3 and 9 min of the temperature shift, respectively [one-way ANOVA, *F*_(5, 20)_ = 10.51, *P* < 0.0001]. As control, resting levels in the incubation medium due to OT release without stimulation was almost unchanged during further seven replacements of incubation medium (see Section Materials and Methods).

To analyze the effect of heat alone, the stimulation sequence was altered, *i.e*., the temperature shift was implemented first followed by the application of cADPR (Figure 1B). In this case, the OT concentration increased by 2.7 ± 0.54-fold relative to the pre-stimulation level (*N* = 21) in response to increasing the incubation temperature from 35 to 38.5°C in the absence of cADPR [one-way ANOVA, *F*_(6, 28)_ = 3.90, *P* < 0.05]. Interestingly, the increase was transient and it was observed during only a fraction of the 3 min period. By contrast, the average increase during 9–15 min in the presence of cADPR together with heat was 3.7 ± 2.7-fold compared with the pre-stimulation level [*N* = 16, one-way ANOVA, *F*_(4, 200)_ = 30, *P* < 0.01]. These transient and accumulated responses with heat and cADPR suggest that heat and cADPR have independent effects on OT release.

The fold increase in the OT concentration induced by cADPR and heat was inhibited significantly with a non-specific TRPM2 inhibitor and a ryanodine receptor antagonist, respectively: In the presence of either 10 μM 2-APB (1.4 ± 0.29-fold relative to the pre-stimulation level, *N* = 7) or 100 μM 8-bromo-cyclic ADP-ribose (8-Br-cADPR; 1.4 ± 0.29-fold relative to the pre-stimulation level, *N* = 7, Figure 1A), respectively [two-way ANOVA, *F*_(12, 78)_ = 6.40, *P* < 0.0001]. Bonferroni's *post hoc* test demonstrated that there was a significant difference between the classes treated with cADPR+heat vs. 2-APB (*P* < 0.001) and cADPR+heat vs. 8-Br-cADPR (*P* < 0.01) at 12 and 15 min. The total increase in OT with different incubation conditions was compared by calculating the total area under the line (TAUL): TAUL_cADPR_ = 34.22; TAUL_8-*Br-cADPR*_ = 20.43; TAUL_2−APB_ = 21.66 arbitrary units, respectively.

In addition, the cADPR- and heat-induced OT concentration increase was not observed in CD38^−∕−^ mice (1.1 ± 0.35, *N* = 8), as shown in Figure 1B [two-way ANOVA, *F*_(6, 56)_ = 3.18, *P* < 0.01]. The difference between the two genotypes was significant [*F*_(1, 56)_ = 40.07, *P* < 0.0001]. Overall, these results suggest that the cADPR- and heat-induced release of OT depends on CD38 and its cADPR-producing enzyme activity as well as TRPM2 cation channels allowing Ca^2+^ influx in the hypothalamus of mice that experienced social stress during group-housing.

### Hypothalamic OT release from mice with CD38 or TRPM2 knockdown *in vitro*

The involvement of CD38 and TRPM2 in OT release was examined pharmacologically in the above experiments. All of the experiments described above were performed using hypothalamus explants from group-housed mice with either the CD38^+∕+^ or CD38^−∕−^ genotype. Thus, the genetic evidence for the involvement of CD38/cADPR is clear, but that for TRPM2 is not. Thus, it would be interesting to perform heat- and cADPR-dependent OT release experiments in TRPM2 KO mice (Uchida et al., 2011) to obtain a clearer understanding of the involvement of TRPM2 in OT release.

However, when we measured the expression levels in the hypothalamus of wild-type C57BL/6 mice, we found that the TRPM2 channel mRNA level was relatively low (Liu et al., 2012). Therefore, we did not perform experiments in TRPM2 KO mice. Instead, we applied the interfering RNA knockdown paradigm to OT release from the hypothalamus.

Lentiviruses with short hairpin RNAs (shRNAs) for CD38 and TRPM2 were injected into the third ventricle of CD38^+∕+^ mice. After 2 weeks of recovery, the CD38 and TRPM2 mRNA levels decreased to 43 ± 3% (*N* = 4) of the scrambled RNA. As expected, the fold increases in the OT concentrations induced by heat stimulation were significantly lower in the media containing the hypothalamus isolated from mice treated with shRNAs for either CD38 (1.6 ± 0.25, *N* = 4) or TRPM2 (1.3 ± 0.43, *N* = 5) compared with those treated with the scrambled shRNA (3.2 ± 1.2, *N* = 3). The fold increases after simultaneous stimulation by heat and cADPR were also significantly lower in mice treated with shRNAs for either CD38 (1.5 ± 0.25, *N* = 6) or TRPM2 (1.3 ± 0.43, *N* = 5) compared with those treated with the scrambled shRNA (3.7 ± 1.2, *N* = 5) [one-way ANOVA, *F*_(2, 11)_ = 4.257, *P* < 0.05]. Thus, these KO and knockdown experiments demonstrated that both CD38 and TRPM2 are involved in the cADPR- and heat-induced facilitation of OT release *in vitro*, although they were not inhibited differentially.

### *In vitro* OT release in mice exposed to social stress

In the above *in vitro* experiments measuring OT release, we observed positive responses but extremely large degrees of variation from one experiment to another. After inspecting the procedure carefully, we noted that no or lower responses were obtained using group-housed wild-type males from the same litters maintained in our animal facility. In addition, the variations in the OT release response were often intensified in group-housed males mixed with different litters from an external supplier.

In the latter housing conditions, fighting was common, and mice were either winners without wounds or losers with wounds. To confirm that the variations in OT release were due to the differences in social status caused by group housing, we applied psychological stress (e.g., exposure to a novel environment; Rasmussen et al., 2011). We determined the hierarchical relationships among mice using a tube test where one mouse forced its opponent to reverse from a narrow tube, thereby allowing us to measure the dominance relations among mice (Lindzey et al., 1961).

Twelve mice from mixed litters were tested in a pairwise manner 14–20 times, where they were assessed on the basis of wining the tests against other mice, and the mice were then ranked into dominant, intermediate, and subordinate groups. The strongest mouse (#1st) was paired with the strongest mouse in the subordinate group (#9th), and similarly the #2nd, 3rd, and 4th mice were paired with the #10th, 11th, and 12th mice in four cages. Cage-mates that lived together received stress by moving them to clean cages every morning for 4 days (cage-switch stress). The amount of OT released was measured in the hypothalamus isolated from one-third of the winning mice (ordinate group) and one-third of the submissive mice (subordinate group).

**Figure 2:**
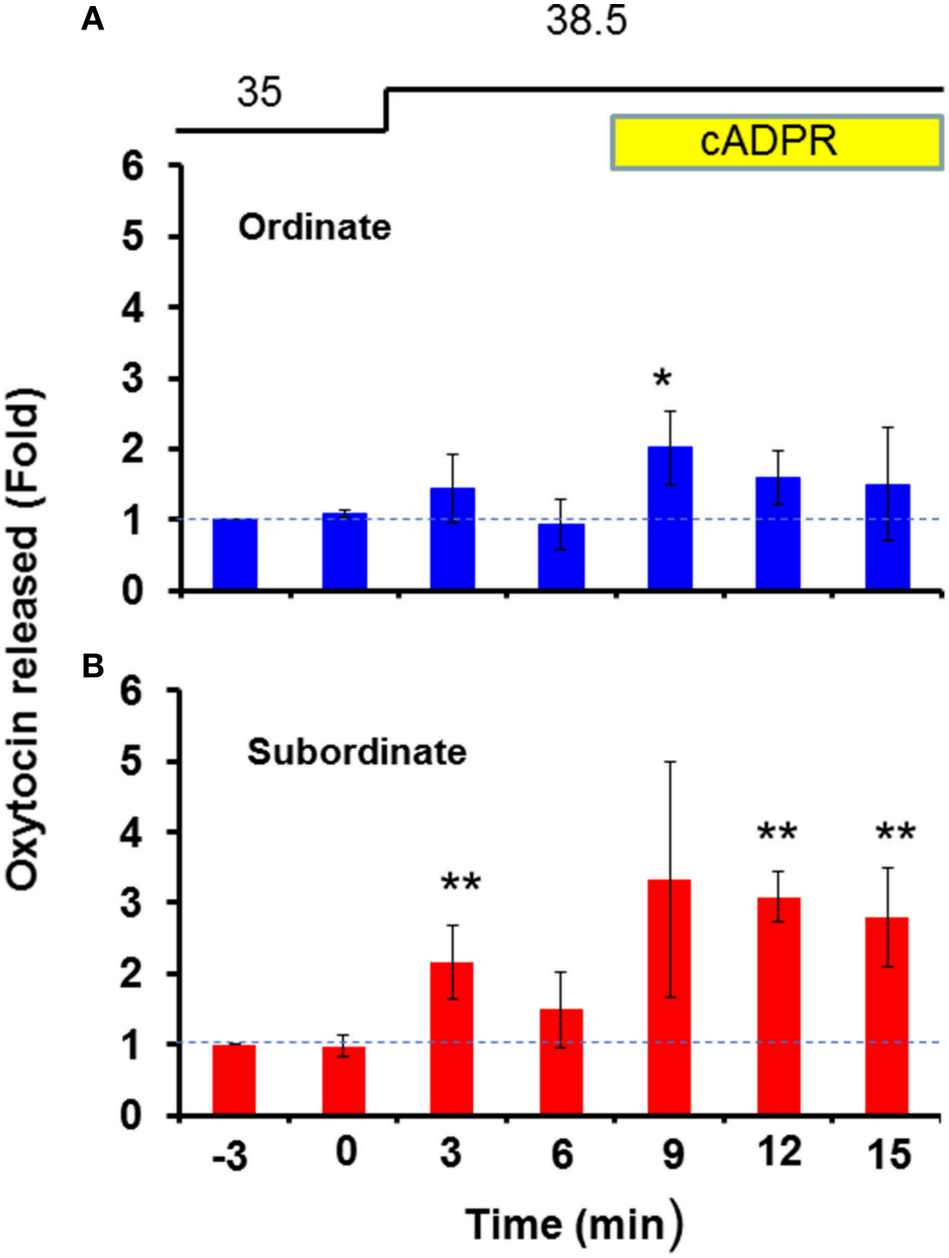
**Oxytocin (OT) release by hypothalamus isolated from ordinate and subordinate mice**. The social status was determined for CD38^+∕+^ mice using the tube test on the 1st day of the experiments, and pairs of one ordinate and one subordinate were housed for 4 days (social stress) with transfer into new cages (cage-switch psychological stress) every day. At 3 h after the last stress, the whole hypothalamus was isolated from ordinate **(A)** and subordinate **(B)** mice. The OT concentration in the incubation medium was measured under stimulation, as shown in Figure 1B (heat followed by cADPR, as indicated). The data are shown as fold changes relative to the OT levels at 6 min before the start of stimulation as 1.0. *N* = 25, one-way ANOVA, *F*_(6, 168)_ = 2.30, *P* < 0.05 in ordinate mice. *N* = 13, one-way ANOVA, *F*_(6, 84)_ = 2.32, *P* < 0.05 in subordinate mice. Bonferroni's *post hoc* tests detected significant differences from the pre-stimulation level at ^*,**^P < 0.05 and 0.01, respectively. The initial value (1.0) at the 12th replacement refers to the concentrations in the incubation medium of 22.3 ± 2.5 and 16.2 ± 7.0 pg/ml for ordinate and subordinate mice, respectively.

As shown in Figure 2, the subordinate group [*N* = 25, one-way ANOVA, *F*_(6, 168)_ = 2.30, *P* < 0.05] released much more OT in response to heat alone or heat and cADPR than the ordinate group [*N* = 13, one-way ANOVA, *F*_(6, 84)_ = 2.32, *P* < 0.05]. The fold increases in the OT concentration in response to heat and cADPR were 1.73 ± 0.64 and 2.08 ± 0.37 (*P* < 0.01) relative to the pre-stimulation level in the subordinate and ordinate mice, respectively. Two-way ANOVA demonstrated that the interaction between treatment and time was not significant [*F*_(6, 56)_ = 0.62, *P* = 0.7162], but there were significant treatment [*F*_(1, 56)_ = 5.02, *P* < 0.05] and time [*F*_(6, 56)_ = 2.54, *P* < 0.05] effects. There was no significant difference between the two groups according to Bonferroni's *post hoc* test. We calculated that TAUL_ordinate_ = 8.45 and TAUL_subordinate_ = 13.13 arbitrary units, respectively.

### *In vivo* OT release by brain perfusion with cADPR in ordinate or subordinate mice

**Figure 3:**
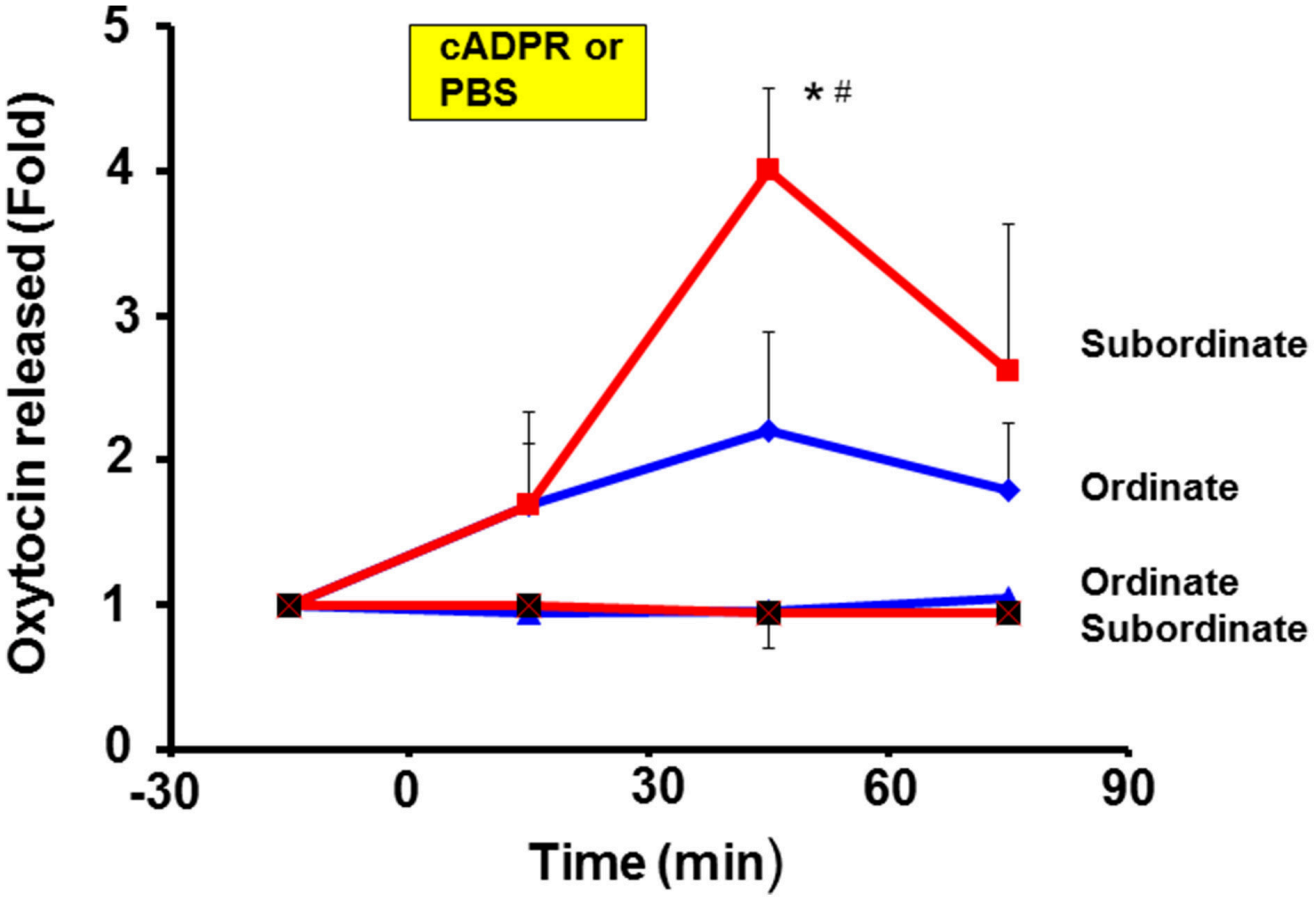
**Elevation of oxytocin (OT) concentrations by cyclic ADP-ribose in brain microperfusates**. After determining the social status using the tube test, ordinate and subordinate pairs were housed (social stress) and exposed to new cages (cage-switch psychological stress) every morning for 4 days. The extracellular fluid of the PVN in both types of mice was collected every 30 min for 30 min using the push-pull type microperfusion system. Data are shown as fold changes in the OT levels for 30 min before the start of stimulation as 1.0. Time courses are shown for subordinate mice with 100 μM cADPR (red squares) or saline (squares with cross, control) and for ordinate mice with cADPR (blue diamonds) and saline (blue triangles, control). One-way ANOVA followed by Bonferroni's *post hoc* test: *N* = 5, *F*_(3, 16)_ = 4.40, *P* < 0.05 for subordinate mice with cADPR; *N* = 6, *F*_(3, 20)_ = 1.13, *P* = 0.3594 for ordinate mice. ^*^P < 0.05 from the basal level. ^#^*P* < 0.01 compared with the values for mice with saline. The analysis of the group results by two-way ANOVA detected significant effects of the treatment and time interaction [*F*_(9, 288)_ = 5.33, *P* < 0.0001]. There were also significant treatment [*F*_(3, 288)_ = 22.53, *P* < 0.0001] and time [*F*_(3, 288)_ = 10.05, *P* < 0.0001] effects. The value (1.0) at the start of sampling (–30 min) refers to the concentration in the perfusate of 136.7 ± 11.5 pg/ml for all 16 mice.

It is necessary to demonstrate OT release from the hypothalamus *in vivo* and to show distinct high or low levels of release in subordinate and ordinate mice, respectively. For the first step, we used the push-pull type of brain microperfusion method under free-moving conditions of pairs of mice that had been exposed to repeated social stress by cage switching, as shown in Figure 2. The amount of OT released over 60 min in the subordinate mice (4.1 ± 0.6-fold, *N* = 5) was significantly greater than that in the ordinate mice (2.2 ± 0.5-fold, *N* = 6) relative to the pre-stimulation level, and compared to the levels in the ordinate or subordinate mice perfused with saline as controls (0.81 ± 0.32-fold vs. 1.22 ± 0.17-fold of the pre-stimulation level, respectively; Figure 3).

The analysis of the group results by two-way ANOVA detected a significant effect of the treatment and time interaction [*F*_(9, 288)_ = 5.33, *P* < 0.0001]. There were also significant treatment [*F*_(3, 288)_ = 22.53, *P* < 0.0001] and time [*F*_(3, 288)_ = 10.05, *P* < 0.0001] effects. These results suggest that cADPR is efficient for releasing OT *in vivo* at normal body temperature (which is already sufficiently high) and/or with neuronal depolarizing activities, but without priming by increasing the body temperature.

### *In vivo* OT release and hyperthermia in mice during exposure to an open field

**Figure 4:**
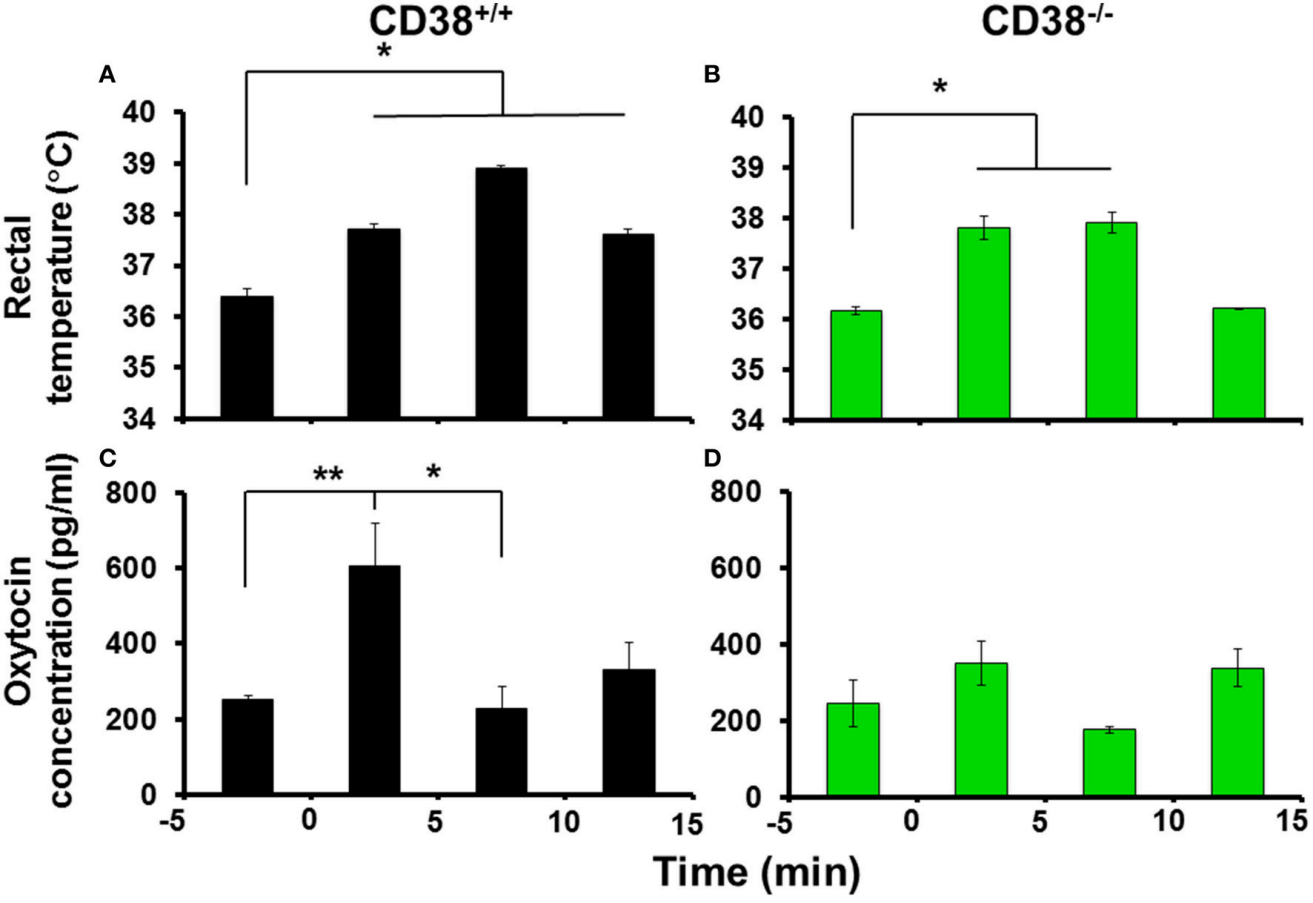
**Body temperature and oxytocin (OT) concentrations in the cerebrospinal fluid (CSF) after exposure to a novel environment stress**. Rectal temperatures of CD38^+∕+^ **(A)** and CD38^−∕−^ **(B)** mice before and after 5, 10, and 15 min of exposure in the open field (anxiety stress). *N* = 9–18, One-way ANOVA *F*_(3, 28)_ = 8.373, *P* < 0.001 in CD38^+∕+^ mice; *N* = 9–18, *F*_(4, 62)_ = 4.60, *P* < 0.005 in CD38^−∕−^ mice. OT concentrations in the CSF collected from CD38^+∕+^ **(C)** or CD38^−∕−^ **(D)** mice exposed to the open field measured over the same time course. One-way ANOVA followed by Bonferroni's *post hoc* tests: *N* = 9–18, *F*_(4, 62)_ = 4.60, *P* < 0.01, ^*,**^P < 0.05 or 0.02, respectively, in CD38^+∕+^ mice; *N* = 5–21, *F*_(4, 50)_ = 1.74, *P* = 0.1567 in CD38^−∕−^ mice.

The most important medical questions in relation to social impairment in psychiatric disorders is whether OT release is associated with hyperthermia in response to social stress, because psychological stress influences behavior and autonomic functions, including hyperthermia (Bouwknecht et al., 2007; Vinkers et al., 2008; Lkhagvasuren et al., 2011). We examined whether social anxiety stress in a new environment could induce OT release due to hyperthermia by exposing mice to the open field where hyperthermia is induced (LeMay et al., 1990). To avoid effects due to group housing stress, in these experiments, we used singly-housed mice that were exposed to the open field. The rectal temperature increased significantly to 37.8°C ± 0.1°C from the control level of 36.4°C ± 0.2°C during the first 5 min, and the temperature increase was maintained for up to 15 min [Figure 4A; *n* = 9–18; one-way ANOVA *F*_(3, 28)_ = 8.373, *P* < 0.001].

The OT concentration in the cerebrospinal fluid (CSF) was also increased at 5 min after exposure in the open field, where the average concentration was 605 ± 114 pg/ml compared with a pre-exposure control level of 251 ± 13 pg/ml [one-way ANOVA followed by Bonferroni's *post hoc* test, *N* = 9–18, *F*_(4, 62)_ = 4.60, *P* < 0.005]. Surprisingly, at 10 and 15 min after exposure, the CSF concentration had already returned to the control level (Figure 4B).

In identical open field stimulation conditions using CD38^−∕−^ mice (Figures 4C,D, *N* = 5–21), the increase in the rectal temperature began during the first 5 min [37.8 ± 0.2°C from 36.6 ± 0.1°C, *P* < 0.01; one-way ANOVA, *F*_(3, 28)_ = 7.733, *P* < 0.0001], but no significant increase in the OT level in the CSF was observed in the identical open field stimulation [one-way ANOVA, *F*_(4, 50)_ = 1.74, *P* = 0.1567]. These results demonstrate that OT release responded transiently during the initial phase of psychological stress (within 5 min) with an increase in body temperature in the CD38^+∕+^ mice, but the increase was not sustained.

### OT concentration in the CSF during hyperthermia in mice treated with lipopolysaccharide (LPS)

**Figure 5:**
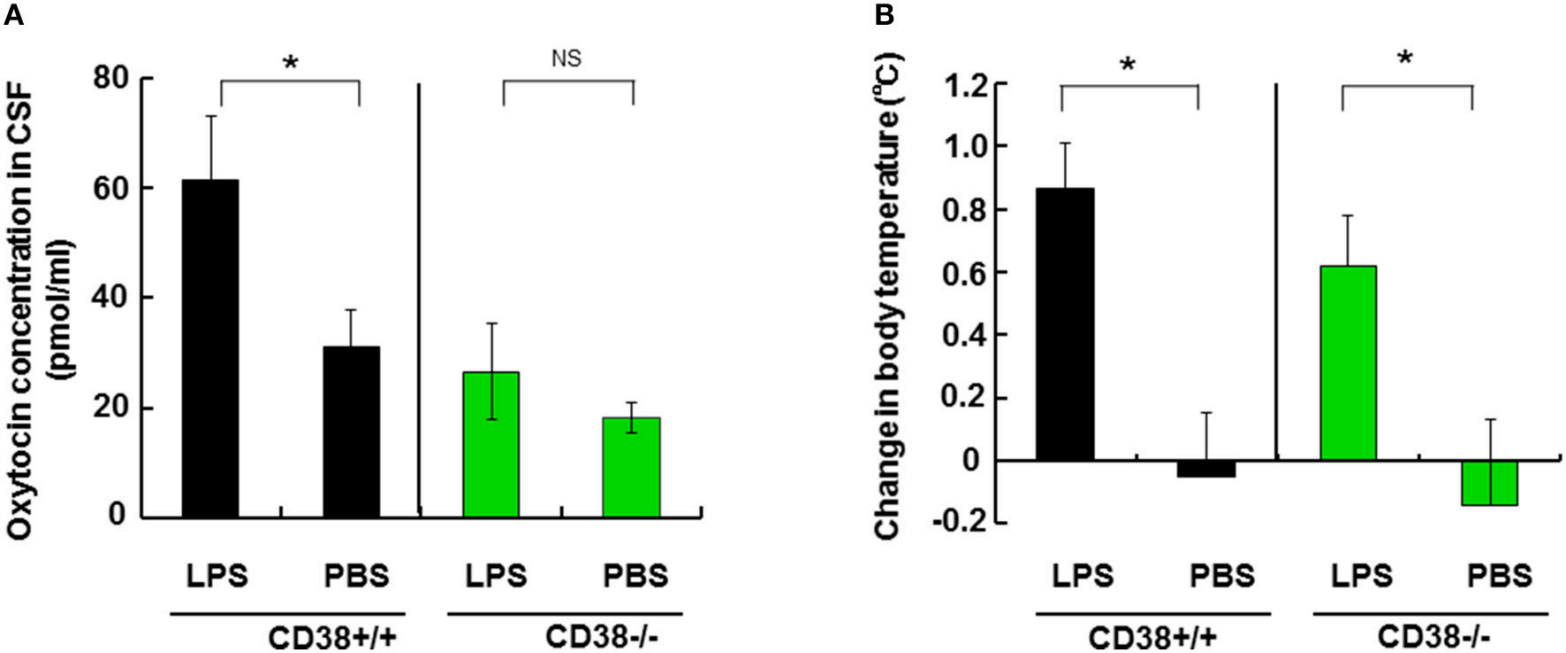
**Effect of lipopolysaccharide (LPS) on the cerebrospinal fluid (CSF) oxytocin (OT) concentration and body temperature. (A)** CSF concentrations of OT in mice at 24 h after treatment by intraperitoneal injection with LPS (3 ng/kg) or saline (PBS) in CD38^+∕+^ or CD38^−∕−^ mice. **(B)** The rectal temperature is expressed as the difference relative to the initial levels (time at 0) in the CD38^+∕+^ or CD38^−∕−^ mice. Two-way ANOVA detected significant treatment [*F*_(1, 16)_ = 8.37, *P* = 0.0106] and genotype [*F*_(1, 16)_ = 5.30, *P* = 0.0351] effects, and Bonferroni's *post hoc* tests detected a significant difference between LPS and PBS treatment in CD38^+∕+^ mice (*P* < 0.0001). There was no significant difference between the treatment and genotype interaction [*F*_(1, 16)_ = 1.84, *P* = 0.1934] in CD38^−∕−^ mice. Two-tailed Student's *t*-test, *N* = 5–10 in each group, ^*^P < 0.05. N.S., not significant. PBS, phosphate-buffered saline.

To obtain further evidence for OT release during hyperthermia, body temperature was manipulated *via* febrile responses in the LPS-induced fever model. Mice and rats exhibit a biphasic body temperature response to LPS: initial hypothermia followed by hyperthermia (Yirmiya et al., 2001). Thus, the rectal temperature in CD38^+∕+^ mice that received intraperitoneal injection of 3 mg/kg LPS decreased during the initial 5–6 h, but it was then maintained at a high level for 15–36 h. At 24 h after injection of LPS, the rectal temperature was 36.2 ± 0.4°C compared with 35.4 ± 0.5°C (*N* = 8) before treatment with an average increase of 0.85 ± 0.14°C (two-tailed Student's *t*-test, *P* < 0.05), whereas the difference between 0 and 24 h was –0.04 ± 0.20°C (*N* = 6) in phosphate-buffered saline (PBS)-treated mice (Figure 5B).

Based on the temperature information, we measured OT concentrations in the CSF that may reflect the effects of hyperthermia on OT release *in vivo*. The OT concentration in the CSF was 60.1 ± 11.6 pg/mg in LPS-treated males, which was double that (30.5 ± 6.6 pg/ml) in PBS-treated control mice (two-tailed Student's *t*-test, *N* = 8, *P* < 0.05).

By contrast, we observed no differences in the CSF OT concentrations of CD38^−∕−^ mice treated with LPS (26.4 ± 9.1 pg/ml) or PBS (18.1 ± 2.5 pg/ml). The rectal temperature increased significantly by 0.62 ± 0.16°C because of LPS treatment in CD38^−∕−^ mice (Figure 5A, *P* < 0.05). Two-way ANOVA detected no significant effect of the treatment and genotype interaction [*F*_(1, 16)_ = 1.84, *P* = 0.1934], but there were significant treatment [*F*_(1, 16)_ = 8.37, *P* = 0.0106] and genotype [*F*_(1, 16)_ = 5.30, *P* = 0.0351] effects. Bonferroni's *post hoc* test detected a significant difference between LPS and PBS treatment in CD38^+∕+^ mice (*P* < 0.0001).

### Mechanisms of facilitative OT release at mRNA and protein levels of CD38 and TRPM2

**Figure 6:**
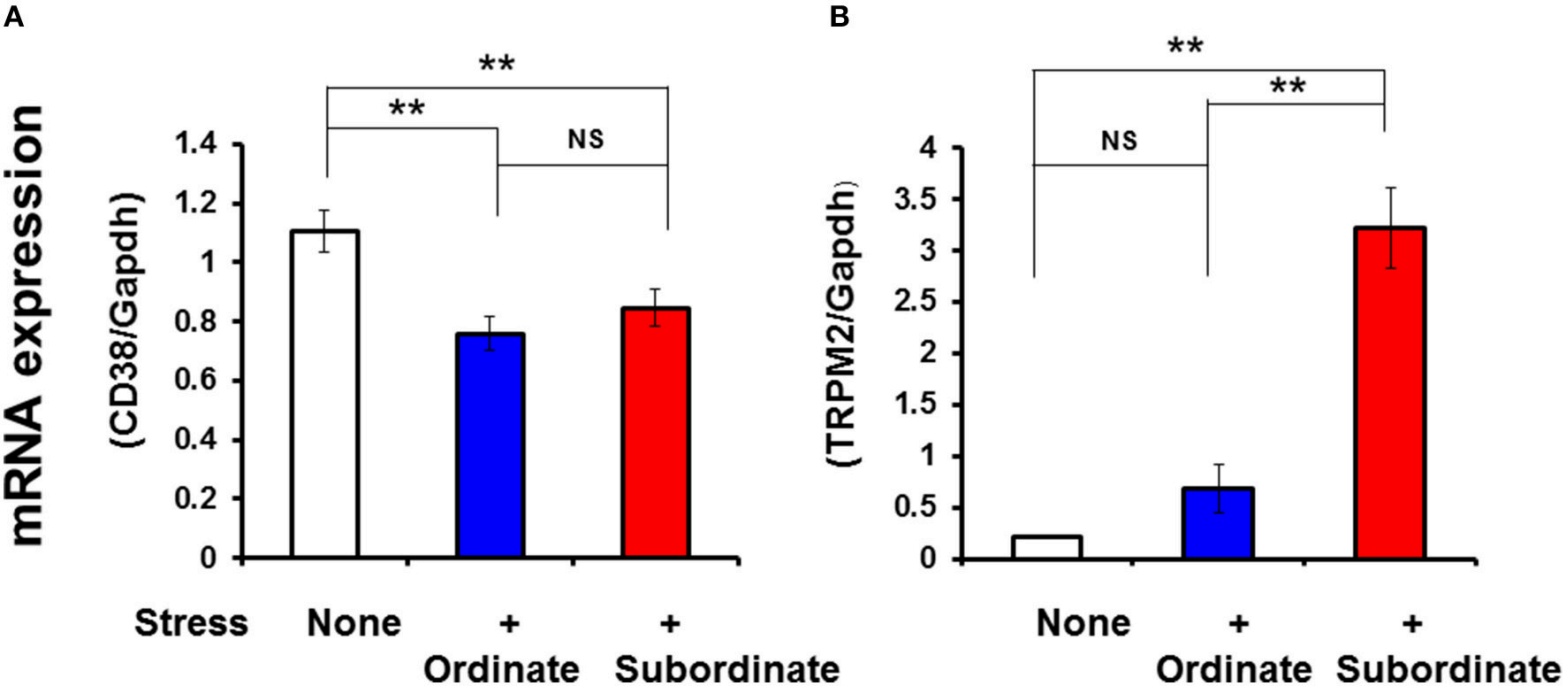
**Social stress-induced alterations in the mRNA expression levels of CD38 and TRPM2**. Total RNA was extracted from the hypothalamus in ordinate and subordinate mice that underwent social and psychological stress by pair-housing and cage-switch for 4 days. As a control, group-housed mice (five mice per cage) were maintained for 4 days without cage-switch stress (open bars). The relative CD38 **(A)** and TRPM2 **(B)** mRNA expression levels (expressed as fold changes relative to the control) in the ordinate (blue) and subordinate (red) groups was determined by relative quantitative RT-PCR with GAPDH as the reference control gene. Two-tailed Student's *t*-test, *N* = 6–7 in each group, ^**^P < 0.01.

The results of the above experiments suggested the contributions of cADPR and hyperthermia for facilitative OT release. Considering the molecular mechanism, it is possible either the kinetic activation or abundance of CD38 and TRPM2 molecules. As it is very difficult to analyze the former possibility, we first analyzed the latter possibility at the mRNA level of CD38 and TRPM2 and the TRPM2 protein (immunoreactivity) level. In the present experiments, the mRNA expression was normalized against that of glyceraldehyde 3-phosphate dehydrogenase (GAPDH) mRNA (Figure 6). The CD38 mRNA levels decreased significantly in the hypothalamus of pair-housed mice with cage-switch stress every morning for 4 days compared with no-stress mice (two-tailed Student's *t*-test, *P* < 0.05, *N* = 4), but there was no difference between the subordinate and ordinate groups.

The TRPM2 mRNA levels increased significantly in the hypothalamus of the subordinate group compared with the ordinate group-housed mice that received the same stress (two-tailed Student's *t*-test, *P* < 0.001, *N* = 4). The TRPM2 mRNA levels in ordinate mice were the same as those of group-housed mice with no stress.

**Figure 7:**
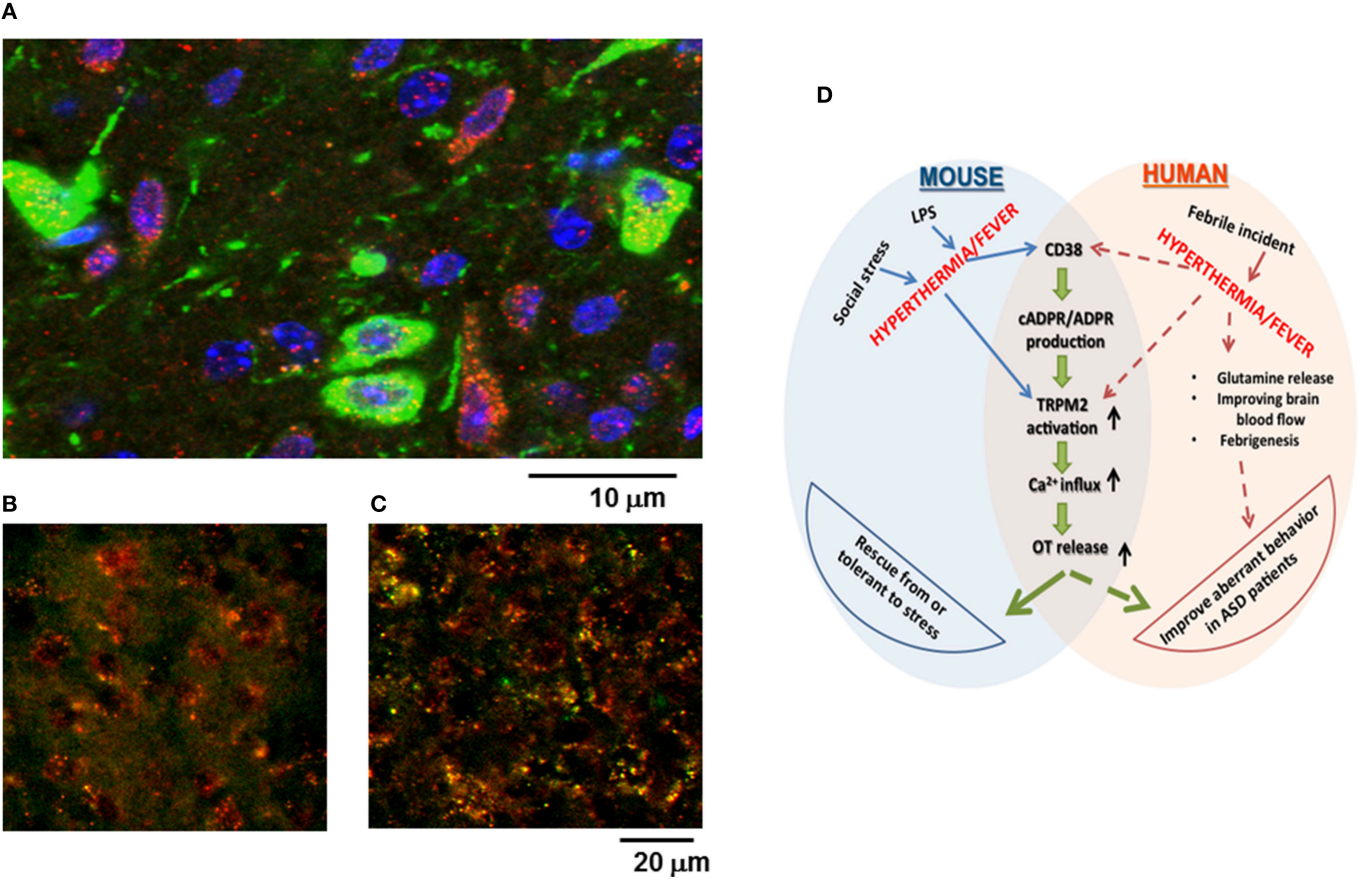
**Co-localization of oxytocin (OT), CD38, or TRPM2 in the paraventricular nucleus (PVN), and a scheme illustrating the relationships among stress, hyperthermia, OT release, and behavior. (A)** The immunoreactivity of the green images represents OT in neuron soma, dendrites, and neuronal fibers. The immunoreactivity of the red images represents TRPM2 and the blue images indicate DAPI (nucleus) in the hypothalamus of group-housed mice. Yellow images in the PVN of the ordinate **(B)** and subordinate **(C)** mice represent the merged immunoreactivity of CD38 (red) and TRPM2 (green). Note that co-localization was intensified in subordinate mice that underwent social and psychological stress with pair-housing and cage-switch. **(D)** This scheme shows the possible molecular mechanisms that underlie stress-induced OT release in the mouse and the proposed pathways involved with improvements in autistic behavior among ASD patients with febrile incidence.

Previously, it was reported that CD38 is highly expressed in the hypothalamus (Jin et al., 2007; Munesue et al., 2010), but it was not shown whether TRPM2 is expressed in the hypothalamus, particularly by oxytocinergic neurons, or how much TRPM2 is co-expressed with CD38. As shown in Figure 7A, TRPM2 immunoreactivity was abundant in the hypothalamus in both oxytocinergic neurons and non-oxytocinergic cells. Co-localization of CD38 and TRPM2 was found in 12.1 ± 3.9% cells (400–500 cells counted in four areas), while TRPM2 and CD38 immunostaining-positive cells comprised 21.1 ± 6.2% and 32.1 ± 4.3% of cells, respectively.

The intensity of immunoreactivity when co-staining for CD38 and TRPM2 appeared to be higher in subordinate mice (Figure 7C) compared with that in ordinate mice (Figure 7B) probably because of the increased intensity of TRPM2.

## Discussion

The release of OT can be regulated by cADPR and heat, which are CD38- and TRPM2-dependent at the protein level. cADPR appears to function by facilitating Ca^2+^ mobilization from intracellular ryanodine-sensitive Ca^2+^ pools and TRPM2 channel gating, because the effect of ADPR on [Ca^2+^]_i_ with heat (at 37°C or 40°C from 35°C in culture medium) was transient and much weaker than the effect of cADPR in NG108-15 neuronal hybrid cells (Amina et al., 2010) and isolated hypothalamic neurons (Liu et al., 2012). To our knowledge, this is the first study of the molecular mechanism underlying OT release from the hypothalamus into the brain showing that both CD38-cADPR and TRPM2-Ca^2+^-influx signals are involved in cellular Ca^2+^ signaling, although the results regarding Ca^2+^ have already been published previously (Amina et al., 2010; Liu et al., 2012).

Alternatively, the release of OT can be regulated by cADPR and heat, which are CD38- and TRPM2-dependent at the protein molecule level. cADPR appears to function by facilitating Ca^2+^ mobilization from intracellular ryanodine-sensitive Ca^2+^ pools and TRPM2 channel gating because the effect of ADPR on [Ca^2+^]_i_ with heat (at 37 or 40°C from 35°C in the culture medium) was transient and much weaker than the effect of cADPR in NG108-15 neuronal hybrid cells (Amina et al., 2010) and isolated hypothalamic neurons (Liu et al., 2012). To the best of our knowledge, this is the first study of the molecular mechanism of OT release from the hypothalamus into the brain to show that both CD38-cADPR and TRPM2-Ca^2+^-influx signals are involved with cellular Ca^2+^ signaling while the results for the Ca part has been already published in two papers (Amina et al., 2010; Liu et al., 2012).

### CSF OT concentrations and stress

The OT concentrations in the CSF increased within 5 min of the start of the psychological stress (anxiety about a new environment) in the open field test. Interestingly, the rectal temperature also increased simultaneously at 5 min from the start of stress exposure, so the increase in the CSF OT concentration appears to have been generated by OT release from the hypothalamus into the brain, which is triggered by hyperthermia and cADPR. However, the time courses of the OT concentration and rectal temperature were quite different, where one was transient and the other was sustained. The transient properties of the increase in the CSF OT concentration appeared to reflect the transient nature of the increases in the OT concentration in the incubation medium induced by the temperature shift alone. Our careful consideration of the previous study by Liu et al. (2012) demonstrated that heat stimulation in the presence of ADPR induced an initial transient increase in [Ca^2+^]_i_, which was as potent as that of cADPR. Thus, the transient release of OT at 5 min in the open field was probably due to the interaction between TRPM2 channels with cADPR and/or ADPR.

Previously, it was reported that changes in emotional behavior (locomotion) in the open field became obvious within a 5 min observation period before the locomotor activity declined to a lower level (Butterweck et al., 2003; Jin et al., 2007). These findings suggest that open field stress effectively controls emotionality within 5 min and that animals adapt gradually to the new environmental stress. According to our observations, the increase in the release of OT occurred at 5 min after exposure to the open field. Thus, it is reasonable to assume that the anxiolytic effect is triggered or at least associated with this OT release.

### Signal pathways leading to OT release

We used CD38^−∕−^ mice or cADPR and TRPM2 channel inhibitors in CD38^+∕+^ mice, but each inhibitor or defect did not discriminate the functional roles of cADPR or TRPM2 in the response. Of course, the concentrations of inhibitors should be considered, but it is possible that these signals may have sequential roles rather than being mediated *via* two independent pathways (Figure 7D).

### Social impairment and autism

To date, there have been several interesting studies of fever in ASD patients. Some autistic children exhibit improvements in their characteristic autistic behaviors during febrile incidents and the regression of fever may be associated with the onset of ASD (Curran et al., 2007; Megremi, 2013; Naviaux et al., 2015). Several possible explanation for this ameliorative effect have been proposed: (1) the release of glutamine from skeletal muscles (Good, 2013); (2) improved brain blood flow (Good, 2011); and (3) febrigenesis and the behavioral state changes associated with fever in autism depend on the selective normalization of key components in a functionally impaired locus coeruleus-noradrenergic system (Mehler and Purpura, 2009), as shown in Figure 7D. However, the OT concentrations have never been considered as the underlying mechanism. In this study, we propose that during a febrific reaction, fever enhances the release of OT to reduce abnormal autistic behavior because direct OT administration improved aberrant behavior in rodents and humans (Jin et al., 2007; Munesue et al., 2010, 2016; Tachibana et al., 2013; Watanabe et al., 2015; Yatawara et al., 2015; Figure 7B).

It has been established that OT plays important roles in social recognition and memory (Insel, 2007; Donaldson and Young, 2008; Carter et al., 2009; Higashida et al., 2011, 2012; Yamasue et al., 2012; Dulac et al., 2014; Rilling and Young, 2014; Numan and Young, 2016; Yamasue, 2016). The KO of OT-related genes such as OT itself (Ferguson et al., 2000), OT receptors (Takayanagi et al., 2005), and the secretory regulator CD38 genes (Jin et al., 2007; Higashida, 2016) lead to social impairment in mice (Modi and Young, 2012; Grinevich et al., 2015) and humans (Meyer-Lindenberg et al., 2011). Recently, accumulating evidence has suggested that single nucleotide polymorphisms in OT, OT receptors, and CD38 genes are associated with autism or high-functioning autism, or they are at least a risk factor (Ebstein et al., 2010; Feldman et al., 2012, 2016; Young and Barrett, 2015). Our results suggest that TRPM2 or single nucleotide polymorphisms, in TRPM2 may be a new target protein and this gene should be screened to assess its association with autism.

## Conclusion

Hyperthermia is likely induced by social stress, as described previously (Singer et al., 1986; Kluger et al., 1987; LeMay et al., 1990; Oka et al., 2003; Adriaan Bouwknecht et al., 2007; Bouwknecht et al., 2007). The social stress procedure has a much more stressful effect on subordinates (social hierarchy; Wang et al., 2014). The results of the present study indicated that larger amounts of OT are released when more stress is experienced, and suggest that, in the subordinate group, the release of more OT seems to allow recovery from stress and toleration of greater stress that will achieve a balance. Finally, TRPM2 may be a new target for modulating social stress and in psychiatric disorders with social impairment.

## Author contributions

HH, H-XL, SAm, OL and SY conceived and designed the research. All performed experiments. OL analyzed data. SAm prepared the initial draft; HH revised the manuscript. All authors reviewed the final manuscript and approved its publication.

### Conflict of interest statement

The authors declare that the research was conducted in the absence of any commercial or financial relationships that could be construed as a potential conflict of interest.

## References

[B1] Adriaan Bouwknecht J. Olivier B. Paylor R. E. (2007). The stress-induced hyperthermia paradigm as a physiological animal model for anxiety: a review of pharmacological and genetic studies in the mouse. Neurosci. Biobehav. Rev. 31, 41–59. 10.1016/j.neubiorev.2006.02.002 16618509

[B2] Amina S. Hashii M. Ma W. J. Yokoyama S. Lopatina O. Liu H. X.. (2010). Intracellular calcium elevation induced by extracellular application of cyclic-ADP-ribose or oxytocin is temperature-sensitive in rodent NG108-15 neuronal cells with or without exogenous expression of human oxytocin receptors. J. Neuroendocrinol. 22, 460–466. 10.1111/j.1365-2826.2010.01978.x 20163520

[B3] Baez D. Raddatz N. Ferreira G. Gonzalez C. Latorre R. (2014). Gating of thermally activated channels. Curr. Top. Membr. 74, 51–87. 10.1016/B978-0-12-800181-3.00003-8 25366233

[B4] Borges B. C. da Rocha M. J. (2006). Participation of the subfornical nucleus in hypothalamic-neurohypophyseal axis activation during the early phase of endotoxic shock. Neurosci. Lett. 404, 227–231. 10.1016/j.neulet.2006.05.052 16815633

[B5] Bouwknecht J. A. Spiga F. Staub D. R. Hale M. W. Shekhar A. Lowry C. A. (2007). Differential effects of exposure to low-light or high-light open-field on anxiety-related behaviors: relationship to c-Fos expression in serotonergic and non-serotonergic neurons in the dorsal raphe nucleus. Brain Res. Bull. 72, 32–43. 10.1016/j.brainresbull.2006.12.009 17303505 PMC1800906

[B6] Brunton P. J. Russell J. A. (2008). The expectant brain: adapting for motherhood. Nat. Rev. Neurosci. 9, 11–25. 10.1038/nrn2280 18073776

[B7] Butterweck V. Prinz S. Schwaninger M. (2003). The role of interleukin-6 in stress-induced hyperthermia and emotional behaviour in mice. Behav. Brain Res. 144, 49–56. 10.1016/S0166-4328(03)00059-7 12946594

[B8] Carter C. S. Boone E. M. Pournajafi-Nazarloo H. Bales K. L. (2009). Consequences of early experiences and exposure to oxytocin and vasopressin are sexually dimorphic. Dev. Neurosci. 31, 332–341. 10.1159/000216544 19546570 PMC2820581

[B9] Curran L. K. Newschaffer C. J. Lee L. C. Crawford S. O. Johnston M. V. Zimmerman A. W. (2007). Behaviors associated with fever in children with autism spectrum disorders. Pediatrics 120, e1386–e1392. 10.1542/peds.2007-0360 18055656

[B10] Donaldson Z. R. Young L. J. (2008). Oxytocin, vasopressin, and the neurogenetics of sociality. Science 322, 900–904. 10.1126/science.1158668 18988842

[B11] Dulac C. O'Connell L. A. Wu Z. (2014). Neural control of maternal and paternal behaviors. Science 345, 765–770. 10.1126/science.1253291 25124430 PMC4230532

[B12] Ebner K. Bosch O. J. Krömer S. A. Singewald N. Neumann I. D. (2005). Release of oxytocin in the rat central amygdala modulates stress-coping behavior and the release of excitatory amino acids. Neuropsychopharmacology 30, 223–230. 10.1038/sj.npp.1300607 15536493

[B13] Ebstein R. P. Israel S. Chew S. H. Zhong S. Knafo A. (2010). Genetics of human social behavior. Neuron 65, 831–844. 10.1016/j.neuron.2010.02.020 20346758

[B14] Faouzi M. Penner R. (2014). TRPM2. Handb. Exp. Pharmacol. 222, 403–426. 10.1007/978-3-642-54215-2_16 24756715

[B15] Feldman R. Monakhov M. Pratt M. Ebstein R. P. (2016). Oxytocin pathway genes: evolutionary ancient system impacting on human affiliation, sociality, and psychopathology. Biol. Psychiatry 79, 174–184. 10.1016/j.biopsych.2015.08.008 26392129

[B16] Feldman R. Zagoory-Sharon O. Weisman O. Schneiderman I. Gordon I. Maoz R.. (2012). Sensitive parenting is associated with plasma oxytocin and polymorphisms in the *OXTR* and *CD38* genes. Biol. Psychiatry 72, 175–181. 10.1016/j.biopsych.2011.12.025 22336563

[B17] Ferguson J. N. Young L. J. Hearn E. F. Matzuk M. M. Insel T. R. Winslow J. T. (2000). Social amnesia in mice lacking the oxytocin gene. Nat. Genet. 25, 284–288. 10.1038/77040 10888874

[B18] Fleming J. O. Ting J. Y. Stohlman S. A. Weiner L. P. (1983). Improvements in obtaining and characterizing mouse cerebrospinal fluid. Application to mouse hepatitis virus-induced encephalomyelitis. J. Neuroimmunol. 4, 129–140. 10.1016/0165-5728(83)90017-6 6300186 PMC7172882

[B19] Franklin K. B. J. Paxinos G. (2008). The Mouse Brain in Stereotaxic Coordinates. Compact, 3rd Edn. New York, NY: Academic Press.

[B20] Good P. (2011). Does fever relieve autistic behavior by improving brain blood flow? Neuropsychol. Rev. 21, 66–67. 10.1007/s11065-011-9157-y 21249454

[B21] Good P. (2013). Does infectious fever relieve autistic behavior by releasing glutamine from skeletal muscles as provisional fuel? Med. Hypotheses 80, 1–12. 10.1016/j.mehy.2012.08.035 23098376

[B22] Grinevich V. Desarménien M. G. Chini B. Tauber M. Muscatelli F. (2015). Ontogenesis of oxytocin pathways in the mammalian brain: late maturation and psychosocial disorders. Front. Neuroanat. 8:164. 10.3389/fnana.2014.00164 25767437 PMC4341354

[B23] Hansen E. W. Christensen J. D. (1992). Endotoxin and interleukin-1 beta induces fever and increased plasma oxytocin in rabbits. Pharmacol. Toxicol. 70, 389–391. 10.1111/j.1600-0773.1992.tb00493.x 1608929

[B24] Hashimoto H. Matsuura T. Ueta Y. (2014). Fluorescent visualization of oxytocin in the hypothalamo-neurohypophysial system. Front. Neurosci. 8:213. 10.3389/fnins.2014.00213 25100939 PMC4107947

[B25] Higashida H. (2016). Somato-axodendritic release of oxytocin into the brain due to calcium amplification is essential for social memory. J. Physiol. Sci. 66, 275–282. 10.1007/s12576-015-0425-0 26586001 PMC4893072

[B26] Higashida H. Yokoyama S. Kikuchi M. Munesue T. (2012). CD38 and its role in oxytocin secretion and social behavior. Horm. Behav. 61, 351–358. 10.1016/j.yhbeh.2011.12.011 22227279

[B27] Higashida H. Yokoyama S. Munesue T. Kikuchi M. Minabe Y. Lopatina O. (2011). CD38 gene knockout juvenile mice: a model of oxytocin signal defects in autism. Biol. Pharm. Bull. 34, 1369–1372. 10.1248/bpb.34.1369 21881219

[B28] Insel T. R. (2007). The challenge of translation in social neuroscience: a review of oxytocin, vasopressin, and affiliative behavior. Neuron 65, 768–779. 10.1016/j.neuron.2010.03.005 20346754 PMC2847497

[B29] Jin D. Liu H. X. Hirai H. Torashima T. Nagai T. Lopatina O.. (2007). CD38 is critical for social behaviour by regulating oxytocin secretion. Nature 446, 41–45. 10.1038/nature05526 17287729

[B30] Kashio M. Tominaga M. (2015). Redox signal-mediated enhancement of the temperature sensitivity of transient receptor potential melastatin 2 (TRPM2) elevates glucose-induced insulin secretion from pancreatic islets. J. Biol. Chem. 290, 12435–12442. 10.1074/jbc.M115.649913 25817999 PMC4424372

[B31] Kato I. Yamamoto Y. Fujimura M. Noguchi N. Takasawa S. Okamoto H. (1999). CD38 disruption impairs glucose-induced increases in cyclic ADP-ribose, [Ca^2+^]_i_, and insulin secretion. J. Biol. Chem. 274, 1869–1872. 10.1074/jbc.274.4.1869 9890936

[B32] Kim U. H. (2014). Multiple enzymatic activities of CD38 for Ca^2+^ signaling messengers. Messenger 3, 6–14. 10.1166/msr.2014.1030

[B33] Kirsch P. (2015). Oxytocin in the socioemotional brain: implications for psychiatric disorders. Dialogues Clin. Neurosci. 7, 463–476. 10.1093/ijnp/pyu012 26869847 PMC4734884

[B34] Kluger M. J. O'Reilly B. Shope T. R. Vander A. J. (1987). Further evidence that stress hyperthermia is a fever. Physiol. Behav. 39, 763–766. 10.1016/0031-9384(87)90263-0 3602130

[B35] Landgraf R. Malkinson T. J. Veale W. L. Lederis K. Pittman Q. J. (1990). Vasopressin and oxytocin in rat brain in response to prostaglandin fever. Am. J. Physiol. 259, R1056–R1062. 2240266 10.1152/ajpregu.1990.259.5.R1056

[B36] Lee H. C. Graeff R. M. Munshi C. B. Walseth T. F. Aarhus R. (1997). Large-scale purification of Aplysia ADP-ribosylcyclase and measurement of its activity by fluorimetric assay. Methods Enzymol. 280, 331–340. 10.1016/S0076-6879(97)80124-3 9211328

[B37] LeMay L. G. Vander A. J. Kluger M. J. (1990). The effects of psychological stress on plasma interleukin-6 activity in rats. Physiol. Behav. 47, 957–961. 10.1016/0031-9384(90)90024-X 2388952

[B38] Leng G. Pineda R. Sabatier N. Ludwig M. (2015). 60 YEARS OF NEUROENDOCRINOLOGY: The posterior pituitary, from Geoffrey Harris to our present understanding. J. Endocrinol. 226, T173–T185. 10.1530/JOE-15-0087 25901040

[B39] Lindzey G. Winston H. Manosevitz M. (1961). Social dominance in inbred mouse strains. Nature 191, 474–476. 10.1038/191474a0 13762409

[B40] Liu H. X. Lopatina O. Amina S. Higashida C. Islam M. S. Graeff R. (2012). Intracellular calcium concentrations regulated by cyclic ADP-ribose and heat in the mouse hypothalamus. Messenger 1, 150–159. 10.1166/msr.2012.1015

[B41] Liu H. X. Lopatina O. Higashida C. Fujimoto H. Akther S. Inzhutova A.. (2013). Displays of paternal mouse pup retrieval following communicative interaction with maternal mates. Nat. Commun. 4:1346. 10.1038/ncomms2336 23299896 PMC4089749

[B42] Liu L. Duff K. (2008). A technique for serial collection of cerebrospinal fluid from the cisterna magna in mouse. J. Vis. Exp. 21:pii960. 10.3791/960 19066529 PMC2762909

[B43] Lkhagvasuren B. Nakamura Y. Oka T. Sudo N. Nakamura K. (2011). Social defeat stress induces hyperthermia through activation of thermoregulatory sympathetic premotor neurons in the medullary raphe region. Eur. J. Neurosci. 34, 1442–1528. 10.1111/j.1460-9568.2011.07863.x 21978215

[B44] Long N. C. Vander A. J. Kunkel S. L. Kluger M. J. (1990). Antiserum against tumor necrosis factor increases stress hyperthermia in rats. Am. J. Physiol. 25, R591–R595. 2316707 10.1152/ajpregu.1990.258.3.R591

[B45] Lopatina O. Liu H. X. Amina S. Hashii M. Higashida H. (2010). Oxytocin-induced elevation of ADP-ribosyl cyclase activity, cyclic ADP-ribose or Ca^2+^ concentrations is involved in autoregulation of oxytocin secretion in the hypothalamus and posterior pituitary in male mice. Neuropharmacology 58, 50–55. 10.1016/j.neuropharm.2009.06.012 19540855

[B46] Lopatina O. Yoshihara T. Nishimura T. Zhong J. Akther S. Fakhrul A. A. K. M.. (2014). Anxiety- and depression-like behavior in mice lacking the *CD157/BST1* gene, a risk factor for Parkinson's disease. Front. Behav. Neurosci. 8:133. 10.3389/fnbeh.2014.00133 24795584 PMC4001052

[B47] Megremi A. S. (2013). Is fever a predictive factor in the autism spectrum disorders? Med. Hypotheses 80, 391–398. 10.1016/j.mehy.2013.01.007 23394936

[B48] Mehler M. F. Purpura D. P. (2009). Autism, fever, epigenetics and the locus coeruleus. Brain Res. Rev. 59, 388–392. 10.1016/j.brainresrev.2008.11.001 19059284 PMC2668953

[B49] Meyer-Lindenberg A. Domes G. Kirsch P. Heinrichs M. (2011). Oxytocin and vasopressin in the human brain: social neuropeptides for translational medicine. Nat. Rev. Neurosci. 12, 524–538. 10.1038/nrn3044 21852800

[B50] Modi M. E. Young L. J. (2012). The oxytocin system in drug discovery for autism: animal models and novel therapeutic strategies. Horm. Behav. 61, 340–350. 10.1016/j.yhbeh.2011.12.010 22206823 PMC3483080

[B51] Morrison S. F. Nakamura K. (2011). Central neural pathways for thermoregulation. Front. Biosci. 16, 74–104. 10.2741/3677 21196160 PMC3051412

[B52] Munesue T. Nakamura H. Kikuchi M. Miura Y. Takeuchi N. Anme T.. (2016). Oxytocin for male subjects with autism spectrum disorder and comorbid intellectual disabilities: a randomized pilot study. Front. Psychiatry 7:2. 10.3389/fpsyt.2016.00002 26834651 PMC4720778

[B53] Munesue T. Yokoyama S. Nakamura K. Anitha A. Yamada K. Hayashi K.. (2010). Two genetic variants of CD38 in subjects with autism spectrum disorder and controls. Neurosci. Res. 67, 181–191. 10.1016/j.neures.2010.03.004 20435366

[B54] Nakayama T. (1985). Thermosensitive neurons in the brain. Jpn. J. Physiol. 35, 375–389. 10.2170/jjphysiol.35.375 2997523

[B55] Naviaux J. C. Wang L. Li K. Bright A. T. Alaynick W. A. Williams K. R.. (2015). Antipurinergic therapy corrects the autism-like features in the Fragile X (Fmr1 knockout) mouse model. Mol. Autism 6:1. 10.1186/2040-2392-6-1 25705365 PMC4334917

[B56] Neumann I. D. Landgraf R. (2012). Balance of brain oxytocin and vasopressin: implications for anxiety, depression, and social behaviors. Trends. Neurosci. 35, 649–659. 10.1016/j.tins.2012.08.004 22974560

[B57] Neumann I. D. Slattery D. A. (2016). Oxytocin in general anxiety and social fear: a translational approach. Biol. Psychiatry 79, 213–221. 10.1016/j.biopsych.2015.06.004 26208744

[B58] Norton E. (2014). Century-old drug reverses signs of autism in mice. Science. Available online at: http://news.sciencemag.org/biology/2014/06/century-old-drug-reverses

[B59] Numan M. Young L. J. (2016). Neural mechanisms of mother-infant bonding and pair bonding: similarities, differences, and broader implications. Horm. Behav. 77, 98–112. 10.1016/j.yhbeh.2015.05.015 26062432 PMC4671834

[B60] Oka T. Oka K. Kobayashi T. Sugimoto Y. Ichikawa A. Ushikubi F.. (2003). Characteristics of thermoregulatory and febrile responses in mice deficient in prostaglandin EP1 and EP3 receptors. J. Physiol. 551, 945–954. 10.1113/jphysiol.2003.048140 12837930 PMC2343282

[B61] Okamoto H. Takasawa S. Sugawara A. (2014). The CD38-cyclic ADP-ribose system in mammals: historical background, pathophysiology and perspective. Messenger 3, 27–34. 10.1166/msr.2014.1032

[B62] Onaka T. Takayanagi Y. Yoshida M. (2012). Roles of oxytocin neurones in the control of stress, energy metabolism, and social behaviour. J. Neuroendocrinol. 24, 587–598. 10.1111/j.1365-2826.2012.02300.x 22353547

[B63] Perraud A. L. Fleig A. Dunn C. A. Bagley L. A. Launay P. Schmitz C.. (2001). ADP-ribose gating of the calcium-permeable LTRPC2 channel revealed by Nudix motif homology. Nature 411, 595–599. 10.1038/35079100 11385575

[B64] Quirin M. Kuhl J. Düsing R. (2011). Oxytocin buffers cortisol responses to stress in individuals with impaired emotion regulation abilities. Psychoneuroendocrinology 36, 898–904. 10.1016/j.psyneuen.2010.12.005 21208748

[B65] Rasmussen S. Miller M. M. Filipski S. F. Tolwani R. J. (2011). Cage change influences serum corticosterone and anxiety-like behaviors in the mouse. J. Am. Assoc. Lab. Anim. Sci. 50, 479–483. 21838975 PMC3148651

[B66] Rilling J. K. Young L. J. (2014). The biology of mammalian parenting and its effect on offspring social development. Science 345, 771–776. 10.1126/science.1252723 25124431 PMC4306567

[B67] Shamay-Tsoory S. G. Abu-Akel A. (2016). The social salience hypothesis of oxytocin. Biol. Psychiatry 79, 194–202. 10.1016/j.biopsych.2015.07.020 26321019

[B68] Singer R. Harker C. T. Vander A. J. Kluger M. J. (1986). Hyperthermia induced by open-field stress is blocked by salicylate. Physiol. Behav. 36, 1179–1182. 10.1016/0031-9384(86)90497-X 3725924

[B69] Stabile A. M. Moreto V. Antunes-Rodrigues J. Carnio E. C. (2010). Central but not systemic inhibition of inducible nitric oxide synthase modulates oxytocin release during endotoxemic shock. Peptides 31, 706–711. 10.3803/EnM.2016.31.2.193 19932725

[B70] Tachibana M. Kagitani-Shimono K. Mohri I. Yamamoto T. Sanefuji W. Nakamura A.. (2013). Long-term administration of intranasal oxytocin is a safe and promising therapy for early adolescent boys with autism spectrum disorders. J. Child Adolesc. Psychopharmacol. 23, 123–127. 10.1089/cap.2012.0048 23480321

[B71] Takasawa S. Nata K. Yonekura H. Okamoto H. (1993). Cyclic ADP-ribose in insulin secretion from pancreatic beta cells. Science 259, 370–373. 10.1126/science.8420005 8420005

[B72] Takayanagi Y. Yoshida M. Bielsky I. F. Ross H. E. Kawamata M. Onaka T.. (2005). Pervasive social deficits, but normal parturition, in oxytocin receptor-deficient mice. Proc. Nat. Acad. Sci. U.S.A. 102, 16096–16101. 10.1073/pnas.0505312102 16249339 PMC1276060

[B73] Togashi K. Hara Y. Tominaga T. Higashi T. Konishi Y. Mori Y.. (2006). Tominaga, M. TRPM2 activation by cyclic ADP-ribose at body temperature is involved in insulin secretion. EMBO J. 25, 1804–1815. 10.1038/sj.emboj.7601083 16601673 PMC1456947

[B74] Tominaga M. Caterina M. J. (2004). Thermosensation and pain. J. Neurobiol. 61, 3–12. 10.1002/neu.20079 15362149

[B75] Uchida K. Tominaga M. (2011). The role of thermosensitive TRP (transient receptor potential) channels in insulin secretion. Endocr. J. 58, 1021–1028. 10.1507/endocrj.EJ11-0130 21785227

[B76] Uchida K. Dezaki K. Damdindorj B. Inada H. Shiuchi T. Mori Y.. (2011). Lack of TRPM2 impaired insulin secretion and glucose metabolisms in mice. Diabetes 60, 119–126. 10.2337/db10-0276 20921208 PMC3012163

[B77] Vinkers C. H. van Bogaert M. J. Klanker M. Korte S. M. Oosting R. Hanania T.. (2008). Translational aspects of pharmacological research into anxiety disorders: the stress-induced hyperthermia (SIH) paradigm. Eur. J. Pharmacol. 585, 407–425. 10.1016/j.ejphar.2008.02.097 18420191

[B78] Wang F. Kessel H. W. Hu H. (2014). The mouse that roared: neural mechanisms of social hierarchy. Trends Neurosci. 37, 674–682. 10.1016/j.tins.2014.07.005 25160682

[B79] Watanabe T. Kuroda M. Kuwabara H. Aoki Y. Iwashiro N. Tatsunobu N.. (2015). Clinical and neural effects of six-week administration of oxytocin on core symptoms of autism. Brain 138, 3400–3412. 10.1093/brain/awv249 26336909

[B80] Yamasue H. (2016). Promising evidence and remaining issues regarding the clinical application of oxytocin in autism spectrum disorders. Psychiatry Clin. Neurosci. 70, 89–99. 10.1111/pcn.12364 26394796

[B81] Yamasue H. Yee J. R. Hurlemann R. Rilling J. K. Chen F. S. Meyer-Lindenberg A.. (2012). Integrative approaches utilizing oxytocin to enhance prosocial behavior: from animal and human social behavior to autistic social dysfunction. J. Neurosci. 32, 14109–141017. 10.1523/JNEUROSCI.3327-12.2012 23055480 PMC6622380

[B82] Yatawara C. J. Einfeld S. L. Hickie I. B. Davenport T. A. Guastella A. J. (2015). The effect of oxytocin nasal spray on social interaction deficits observed in young children with autism: a randomized clinical crossover trial. Mol. Psychiatry. 10.1038/mp.2015.162. [Epub ahead of print]. 26503762 PMC4995545

[B83] Yirmiya R. Pollak Y. Barak O. Avitsur R. Ovadia H. Bette M.. (2001). Effects of antidepressant drugs on the behavioral and physiological responses to lipopolysaccharide (LPS) in rodents. Neuropsychopharmacology 24, 531–544. 10.1016/S0893-133X(00)00226-8 11282253

[B84] Young L. J. Barrett C. E. (2015). Can oxytocin treat autism? Science 347, 825–826. 10.1126/science.aaa8120 25700501 PMC4362686

[B85] Zhao Y. J. Lam C. M. Lee H. C. (2012). The membrane-bound enzyme CD38 exists in two opposing orientations. Sci. Signal. 5:ra67. 10.1126/scisignal.2002700 22969159

[B86] Zhong J. Liang M. Akther S. Higashida C. Tsuji T. Higashida H. (2014). c-Fos expression in the paternal mouse brain induced by communicative interaction with maternal mates. Mol. Brain 7:66. 10.1186/s13041-014-0066-x 25208928 PMC4172782

